# Predictive urinary biomarkers for steroid-resistant and steroid-sensitive focal segmental glomerulosclerosis using high resolution mass spectrometry and multivariate statistical analysis

**DOI:** 10.1186/1471-2369-15-141

**Published:** 2014-09-02

**Authors:** Shiva Kalantari, Mohsen Nafar, Dorothea Rutishauser, Shiva Samavat, Mostafa Rezaei-Tavirani, Hongqian Yang, Roman A Zubarev

**Affiliations:** 1 Department of Basic Sciences, Faculty of Paramedical Sciences, Shahid Beheshti University of Medical Science, Tehran, Iran; 2 Chronic Kidney Disease Research Center, Shahid Beheshti University of Medical Sciences, Tehran, Iran; 3 Department of Nephrology, Shahid Labbafinejad Medical Center, Shahid Beheshti University of Medical Science, Tehran, Iran; 4 Urology and Nephrology Research Center, Shahid Beheshti University of Medical Sciences, Tehran, Iran; 5 Department of Medical Biochemistry and Biophysics, Karolinska Institute, Stockholm, Sweden; 6 SciLifeLab, Stockholm, Sweden; 7 Proteomics Research Center, Shahid Beheshti University of Medical Science, Tehran, Iran

**Keywords:** Urine proteomics, Inflammatory response, Responsiveness

## Abstract

**Background:**

Focal segmental glomerulosclerosis (FSGS) is a glomerular scarring disease diagnosed mostly by kidney biopsy. Since there is currently no diagnostic test that can accurately predict steroid responsiveness in FSGS, prediction of the responsiveness of patients to steroid therapy with noninvasive means has become a critical issue. In the present study urinary proteomics was used as a noninvasive tool to discover potential predictive biomarkers.

**Methods:**

Urinary proteome of 10 patients (n = 6 steroid-sensitive, n = 4 steroid-resistant) with biopsy proven FSGS was analyzed using nano-LC-MS/MS and supervised multivariate statistical analysis was performed.

**Results:**

Twenty one proteins were identified as discriminating species among which apolipoprotein A-1 and Matrix-remodeling protein 8 had the most drastic fold changes being over- and underrepresented, respectively, in steroid sensitive compared to steroid resistant urine samples. Gene ontology enrichment analysis revealed acute inflammatory response as the dominant biological process.

**Conclusion:**

The obtained results suggest a panel of predictive biomarkers for FSGS. Proteins involved in the inflammatory response are shown to be implicated in the responsiveness. As a tool for biomarker discovery, urinary proteomics is especially fruitful in the area of prediction of responsiveness to drugs. Further validation of these biomarkers is however needed.

## Background

Focal segmental glomerulosclerosis (FSGS) is a glomerular scarring disease characterized by increased extracellular matrix within the glomerular tuft [1,2]. The sclerotic lesions occur focally and in only some segments of glomeruli, and are typically not associated with immune complex deposition [1]. It is believed that the loss of foot process of podocytes is also one of the most important characteristics of FSGS [3]. FSGS is a major cause of steroid resistant nephrotic syndrome, which could lead to end-stage renal failure [4,5]. FSGS is currently diagnosed mostly by kidney biopsy [1,2]. Pathological evaluation of renal cortical tissue using renal biopsy has become a traditional way to distinguish between steroid sensitive and steroid resistant patients, since there are no clinical or biochemical parameters for distinguishing between them [6,7]. Owing to the invasive nature and potential complications of biopsy and also lack of absolute correlation between histological findings and patients’ clinical response to treatment [8], identification of noninvasive biomarkers would be most beneficial for steroid-resistant patients, who would avoid exposure to high-dose, yet ineffective steroid courses.

It is therefore essential to search for noninvasive biomarkers for the prediction of steroid responsiveness of glomerular diseases, especially with FSGS as a dominant cause of steroid-resistant nephrotic syndrome. Urine is the most appropriate fluid sample for biomarker discovery in glomerular diseases [9]. Proteomics, with its high-throughput capabilities, is the optimal technique for searching predictive biomarkers in urine. Although large efforts have been made in this field in recent years [10,11], the emergence of the latest high-resolution mass spectrometry tools promises deeper and more accurate analysis that should provide more reliable biomarkers and possibly even shed some light on the disease mechanism.

In this study, we applied urine proteomics using a combination of nanoflow liquid chromatography and a high resolution mass spectrometer to identify biomarkers predictive of FSGS patient responsiveness to steroid therapy. Multivariate statistical analysis was employed to identify the relevant proteins and build a predictive model, and gene ontology enrichment and upstream regulator analysis provided insight into FSGS-related biological processes. However, since a relatively small cohort was used in this pilot study, further analysis of a larger cohort is required to validate these findings.

## Methods

### Patients

At Labbafinejad Hospital, 10 patients (six steroid sensitive and four steroid resistance) with biopsy proven FSGS were consecutively enrolled in this study during 2011. The samples were coded to protect confidentiality. Age, sex, smoking habits and also diet (a day before sampling) of the patients were noted and patients with other implications like diabetes were excluded. All patients agreed to the use of their urine samples for this study. Written consents were given by the participants according to the recommended procedure and consent form provided by the medical ethics committee of the Shahid Beheshti university of medical sciences. The consent forms signed by each participant signed included the following issues: brief introduction of the study, its benefit to research, potential dangers, confidentiality of the identity of participants, contact information for answering eventual questions, and the right to resign from the study. The Medical ethics committee of Shahid Beheshti university of Medical sciences approved this consent forms as well as this study (date of approval: 12/17/2010). For each patient, data were collected concerning serum creatinine, eGFR (by CKD-EPI equation), presence of hypertension and proteinuria at presentation. All the patients had same the race/ethnicity.

The patients were treated with prednisolone 1 mg/kg for 6 to 8 weeks and 24 hours urine samples were collected for evaluation of the response to treatment. Complete remission was defined as urine protein excretion less than 200 mg per day and partial response is referred to a 50% reduction in proteinuria. While KDIGO guidelines define steroid-resistance in adults as presence of proteinuria despite high-dose prednisolone for >4 months, we consider 16 weeks of high-dose therapy too long a period that may negatively affect nonresponding patients. According to the policy of our Center, if a patient shows no response to high prednisolone in 8 weeks, the immunosuppressive therapy protocol must be changed to avoid the side effects of high dose steroids.

### Sample preparation and protein extraction

All the urine samples were taken at the time of diagnosis and before the initiation of treatment. Approximately 20–40 mL of second morning midstream urine from patients were collected from the subjects. Urine samples were immediately put on ice and 1 mL of dissolved protease inhibitor (one tablet, Cocktail protease inhibitor, Sigma, dissolved in distilled water) was added to each 10 mL of urine. The samples were centrifuged at 3000 rcf for 20 minutes at 4°C to pellet the cell debris. The supernatant was divided and transferred into 15 mL tubes and stored at -80°C until the samples were processed further. The supernatant was concentrated and desalted by ultrafilteration as follow: urine samples were transferred to individual Amicon Ultra-15 Centrifugal Filter Units with a 3 kDa cutoff (Millipore, Billerica, MA, USA) and spun at 3220 rcf at 4°C for 1 h. The initial concentration was followed by two wash steps by adding 14 mL of PBS and spinning each tube at 3220 rcf at 4°C for 1 h. By filtering, the sample volume was reduced from 15 mL to a final volume of approximately 800–1000 μL. In order to inactivate potential bacterial activity, 1200 μL of cold acetone was added to 300 μL of concentrated urine and incubated at -20°C overnight. The samples were dried in a vacuum concentrator and stored at -20°C.

Dried samples were re-suspended in 0.1 M ammonium acetate (pH 5) and the protein concentrations of the samples were determined using the BCA (Bicinchoninic Acid) Protein Assay (Pierce, Thermo Scientific, USA). 10 μg urinary proteins from the individual samples were resolved in a buffer containing 0.1% ProteaseMax, 10% acetonitrile, 1 M urea and 50 mM ammonium bicarbonate and digested in duplicates using sequencing-grade trypsin (Promega, USA). Samples were then reduced with 20 mM DTT (dithiothreitol) for 30 min at 56°C and alkylated with 66 mM IAA (Iodoacetamide) for 30 min at room temperature in the dark. The samples were digested by trypsin in a ratio of 1:50 (enzyme: protein) at 37°C overnight. The resulting peptides were desalted using C18 StageTip (Thermo Scientific, USA). The eluted peptides were evaporated in a SpeedVac and re-suspended in a buffer containing 0.1% formic acid and 3% ACN (acetonitrile) v/v before loading to a nano-LC-MS/MS system.

### Liquid chromatography tandem mass spectrometry

Liquid chromatography tandem mass spectrometry (nLC-MS/MS) analyses were performed on an Easy-nLC system coupled online to a Q Exactive mass spectrometer (both - Thermo Scientific, Bremen, Germany). Separation of peptides was performed using a 10 cm fused silica tip column (SilicaTips™ New Objective Inc., Woburn MA, USA) in-house packed with Reprosil-Pur C18-AQ 3 μm resin (Dr. Maisch GmbH, Ammerbuch-Entringen, Germany) using a methanol slurry and a pressurized “packing bomb” operated at 40 bar (Proxeon Biosystems). Mobile phases consisted of 0.1% formic acid in water v/v (buffer A) and 0.1% formic acid in acetonitrile v/v (buffer B). The LC gradient was set up as following: 5 - 35% buffer B in 89 min, 48 - 80% buffer B in 5 min, and 80% buffer B for 8 min, all at a flow rate of 300 nL/min. Samples (10 μL corresponding to approximately 2.3 μg of total protein) were injected via a temperature-controlled autosampler.

The MS acquisition method was comprised of one survey full scan ranging from m/z 300 to m/z 1650 acquired with a resolution of R = 70,000 at m/z 400, followed by data-dependent HCD (higher energy collision dissociation) MS/MS of maximum ten most abundant precursor ions with a charge state ≥ 2. MS/MS spectra were acquired with a resolution of R = 17,500, with a target value of 2e5 ions, isolation m/z width was set to 4 and normalized collision energy to 26 eV. For all sequencing events, dynamic exclusion was enabled and unassigned charge states were rejected. The instrument was calibrated externally according to the manufacturer’s instructions and all data were acquired using internal lock mass calibration on m/z 429.088735 and 445.120025 (background ions). All the experiments were done in duplicate (20 runs in total).

### Protein identification and quantification

Tandem mass spectra were extracted using Raw2MGF (in-house-written program) and the resulting Mascot generic files (.mgf) were searched against a concatenated SwissProt protein database (Human taxonomy) using Mascot 2.3.0 search engine (Matrix Science Ltd., London, UK). Carbamidomethylation of cysteine was set as a fixed modification and deamidation of asparagine and glutamine as well as oxidation of methionine were set as variable modifications. Up to two missed tryptic cleavages were allowed and the mass tolerance was set to 10 ppm and to 0.05 Da for the precursor and fragment ions, respectively. Only peptides having individual MS/MS Mascot score above significant threshold corresponding to E < 0.05 were accepted. Only proteins identified with at least two unique peptides with a significant score and at 0.25% false discovery rate (FDR) were considered for further quantification.

Relative abundance of these proteins was determined using Quanti software (an in-house developed software package [12]), which performs accurate label-free peptide and protein quantification with correction for instrumental response fluctuations. The areas of the chromatographic peaks were taken as the peptide abundances and the same peptides were quantified in each nLC-MS/MS data file using accurate mass and the order of elution as identifiers. The sum of the abundances of all unique peptides of a protein was used as the protein abundance value. The list of quantified proteins was further filtered to 1% FDR, which corresponded to the protein Mascot score of 23.73.

### Statistical analysis

Prior to statistical analysis, the data were transformed (log2) in order to make normal distribution of data which is a prerequisite for parametric statistical tests and deleted all the proteins with at least one missing value. The quantitative proteome data was subjected to multivariate statistical analysis using SIMCA (SIMCA-p 13.0, Umetrics, Umeå, Sweden). The protein abundances from each replicate analysis were used as the primary variables and 10 patient samples were considered as primary observations. The analysis was performed on unit variance scaled data, assuming equal importance of each protein regardless of their relative abundance and magnitude of variance between the samples. Unsupervised principal component analysis (PCA [13,14])) was performed for detecting the clusters of the data if any and identifying statistical outliers.

PCA explains the variance-covariance structure of a set of variables by using linear combinations of those [15]. The linear combination represents a new coordinate system that is obtained by rotating the original variable space. The new axes acquired will represent the directions with maximum variability where PC1 indicates the direction of the highest variability followed by consecutive PCs with diminishing variability orthogonal to the previous PC or PC plane [16]. For classification and identification of proteins separating steroid resistant from steroid sensitive patients, we used orthogonal projection to latent structures discriminant analysis (OPLS-DA) [17]. The OPLS analysis detects the proteins whose abundances covary with the defined clinical group. OPLS-DA model was built for discrimination of responder and non-responder groups. To avoid overestimation, sevenfold cross-validated scores were calculated [18]. These scores were used for receiver operating characteristic (ROC) analysis to estimate the predictive accuracy of the model [19]. As a negative control, responder/non-responder identifier was randomized for all samples, and the above procedure was repeated.

### Protein GO-term enrichment and regulator analysis

For general characterization of properties of the proteins in the data set and also detection of the enriched cellular component, molecular function and biological process, gene ontology enrichment was performed using DAVID open-source software tool [20]. DAVID uses the EASE score [21], a modified Fisher Exact p-value, to determine whether a GO-term is over- or under-represented in a given proteomic data set with reference to a background data set (e.g. the human proteome). A cut off for enrichment score for DAVID software result was set at 1.3 and the redundant hits were excluded.

## Results

### Clinical and pathological characteristics of patients

Clinical and laboratory information on patients is provided in Table 1. Ten patients (six males and four females, aged between 18–61 years, mean age 37.1 years) with biopsy-proven FSGS were enrolled. A single pathologist reviewed the biopsy samples. In order to estimate the amount of protein excretion, 24-hour urine collection was used. The mean 24-hour protein concentration was 3720 mg/day. Renal function was evaluated by eGFR using CKD-EPI equation. The mean eGFR level was 63.6 cc/min/1.73 m^2^ and five patients had eGFR less than 60 cc/min/1.73 m^2^ at presentation. All the patients were HIV negative. No morbidly obese patients were included. There was no evidence of secondary FSGS on pathology samples. The p-values for the relationship between clinical features and responsiveness were not significant: for age (p-value = 0.34); eGFR (p-value =0.21); proteinuria (p-value =0.7); TA/IF (p-value =0.09); global glomerulosclerosis (p-value =0.9).

**Table 1:** Demographic and laboratory characteristics of patients with focal segmental glomerulosclerosis

**Patient’s code**	**Age (yr)**	**Sex**	**eGFR (cc/min/1.73 m**^**2**^**)**	**Proteinuria (mg/day)**	**TA/IF**	**Global glomerulosclerosis**	**Responsiveness**
1	29	M	34.61	2031	<10%	0%	Partial Responder
2	46	M	34.64	5000	30%	30%	Non-responder
3	19	M	145.76	4500	30%	23%	Partial Responder
4	61	M	46.52	2590	<10%	16%	Partial Responder
5	37	F	78.51	1400	<10%	8%	Partial Responder
6	36	F	60.52	2710	20%	26%	Partial Responder
7	37	F	42.01	710	30%	30%	Non-responder
8	30	M	38.76	2925	40%	45%	Non-responder
9	58	F	70.48	4373	<10%	0%	Non-responder
10	18	M	85.17	11000	<10%	0%	Complete Responder

(eGFR: Estimated Glomerular Filtration Rate by CKD-EPI equation, TA: tubular atrophy, IF: interstitial fibrosis). All the partial responders were included in the responder group.

### Proteomics analysis

After false discovery rate (FDR) assessment (≤1%) using in house-written software which was also used for quantification [12], 368 protein entries, each with ≥2 unique peptides, were quantified (Additional file 1). The data set after preprocessing procedure reduced to 110 protein entries (Additional file 2).

### Unsupervised statistical analysis by PCA

**Figure 1:**
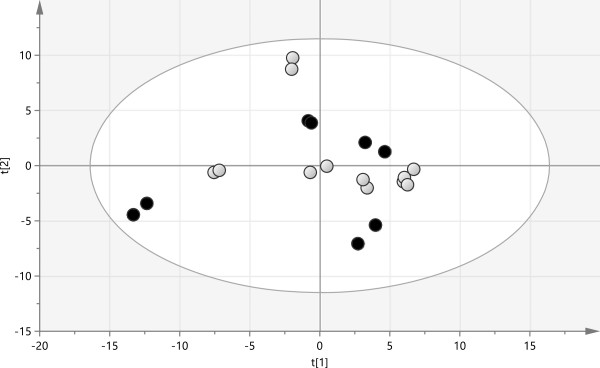
**Score plot of PCA.** Open circles represent steroid sensitive (responder) and black dots represent steroid resistant (non-responder) patient samples. Each of the 10 samples was analyzed with two technical replicates.

The dataset produced a single cluster in two-dimensional PCA, without any outlier (Figure 1). The PCA coordinates did not correlate with patients’ sensitivity to treatment, sex or any other known to us baseline parameter or clinical characteristics. The lack of clustering in PCA score plot means that there was no confounding factor that affected our study, and that the difference between responders’ and non-responders’ proteomes was relatively small. Therefore, supervised orthogonal projection to latent structures discriminant analysis (OPLS-DA) was used to reveal these differences.

### Supervised statistical analysis by OPLS-DA

**Figure 2:**
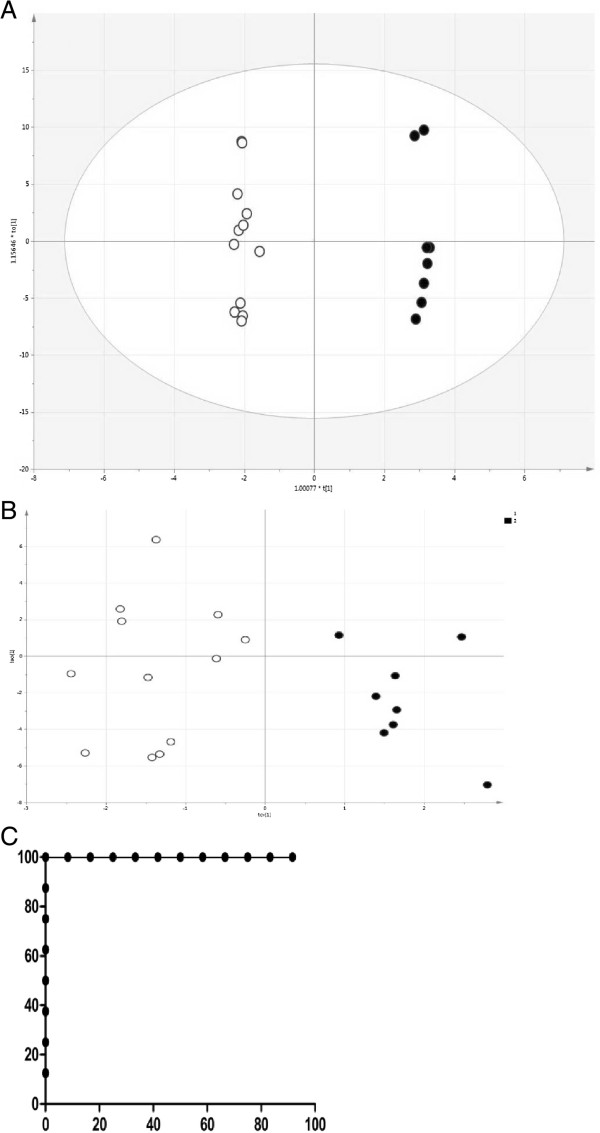
**Predictive model. A)** Orthogonal projection to latent structures discriminant analysis (OPLS-DA) model for discrimination of steroid sensitive (open circles) and steroid resistant (black dots) patient samples. **B)** Separation of the same samples by a seven-fold cross validated model built based on OPLS-DA in A); **C)** ROC-curve based on model in B).

**Figure 3:**
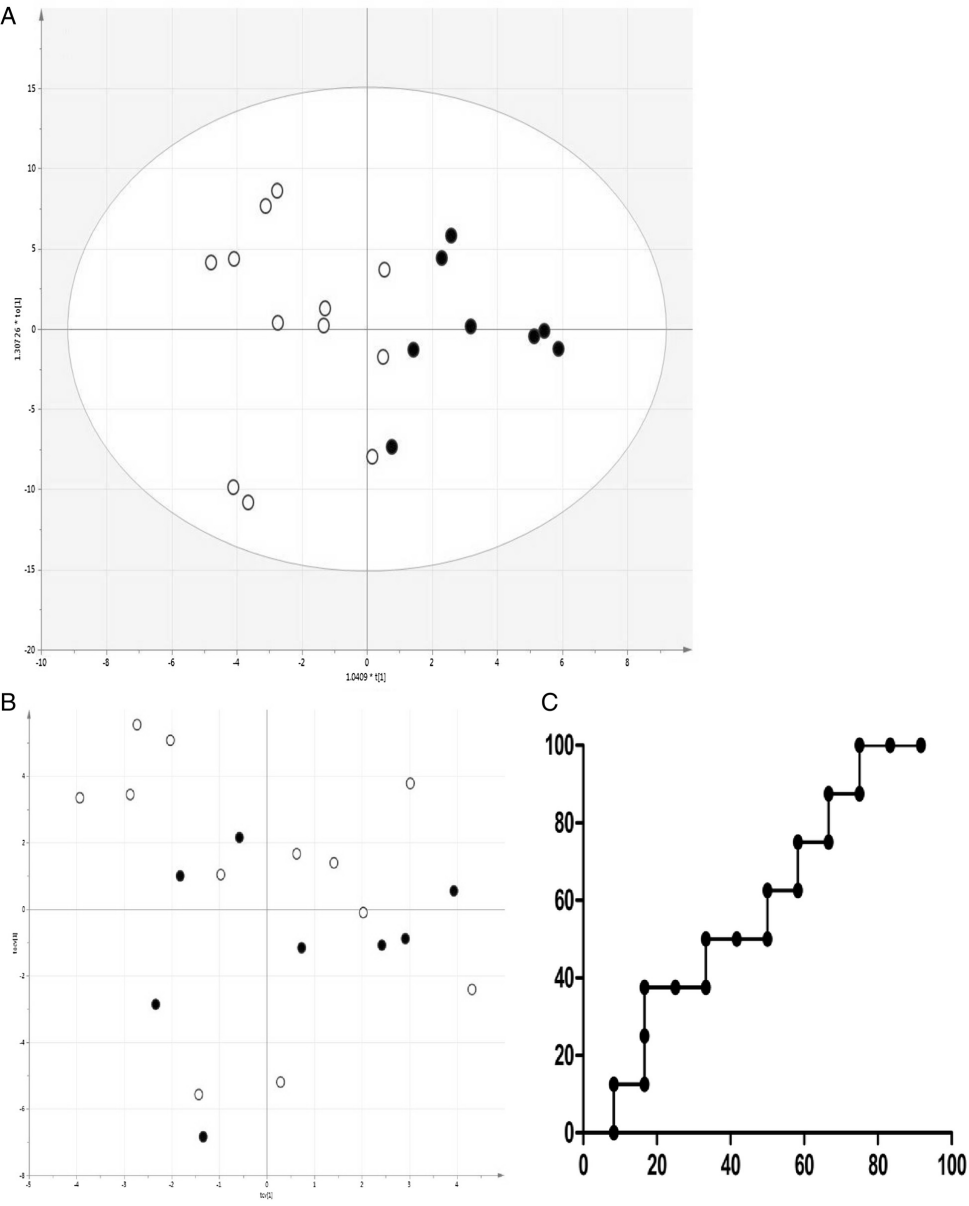
**Randomized model. A)** OPLS-DA model for the decoy discrimination of steroid sensitive (open circles) and steroid resistant (black dots) patient samples. **B)** Separation of the same samples by a seven-fold cross validated model built based on OPLS-DA in A); **C)** ROC-curve based on model in B).

110 proteins were normalized in both groups with FDR ≤ 1% and the other requirements described above (Additional file 2). OPLS-DA model gave R2 = 0.996 and Q2 = 0.756 for the discrimination of responder and non-responder groups, and showed the predictive accuracy of 100% (Figure 2). 21 proteins significantly contributing to the responder/non-responder discrimination by the OPLS-DA model. These proteins are listed in Additional file 3 with their average responder/non-responder abundance ratio for each protein. 13 proteins were underrepresented and eight proteins overrepresented in responders compared to non-responders.In the negative control, where responder/non-responder identifiers were randomized for all samples, no valid OPLS-DA model (R2 = 0.694, Q2 = -0.216) was obtained, and cross-validation confirmed that the model is statistically indistinguishable from random guessing with the area under the curve (AUC) of only 0.59 (Figure 3). This negative result substantiates the validity of the cross-validated predictive model on Figure 2.

### GO-term enrichment analysis

**Figure 4:**
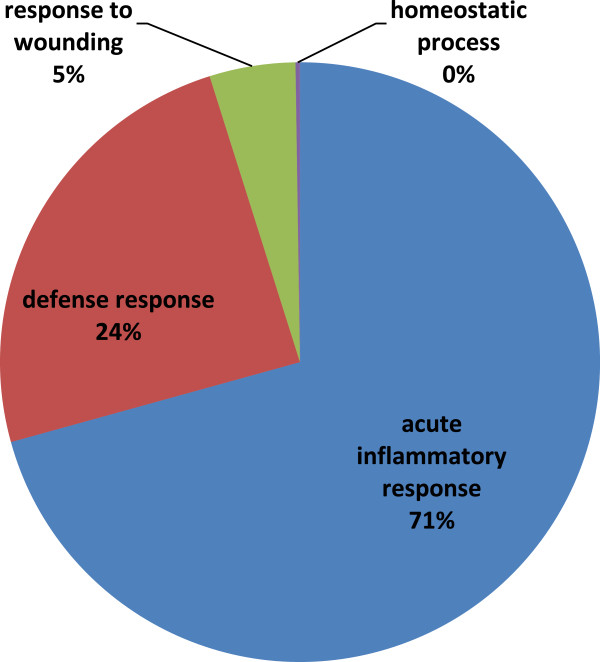
**Gene set enrichment analysis of biological process.** This analysis was done by DAVID based on the predictive proteins in Additional file 3.

Gene ontology enrichment analysis of the 21 most differentiating proteins obtained from the predictive OPLS-DA model, using DAVID software revealed “acute inflammatory response” (p-value = 1. 9 × 10^-4^), “defense response” (p-value = 5.5 × 10^-4^), “response to wounding” (p-value = 2.9 × 10^-3^) and “homeostatic process” (p-value =5.6 × 10^-2^) as the most significant biological processes (Figure 4) (see the details in Additional file 4). In pathway analysis, no enriched signaling or metabolic pathway was found.

## Discussion

A noninvasive biomarker from urine sample that predicts responsiveness to steroid therapy in focal segmental glomerulosclerosis would be valuable for choosing the correct therapeutic strategy which should lead to reduced time of healing.

Urine proteomics can be utilized to identify a panel of biomarkers associated with steroid resistance in FSGS. We performed nLC-MS/MS using high resolution mass spectrometer (Q-Exactive) and used supervised multivariate statistical analysis to identify proteins differentiating the two groups. In the list of the identified candidates, some proteins have been reported before, but most molecules are novel and thus need to be further validated on a larger cohort. Relationship between some of these biomarkers and responsiveness to steroid therapy as well as glomerular sclerosis is discussed below.

The most significantly overrepresented protein in the steroid sensitive group (responders) was APOA1 (apolipoprotein A-1) (fold change = 3.15). Kunitake et al. have found in 1992 that all transition metal ions binding to lipoprotein (A-I) (which contains APOA1) are known ligands of transferrin or ceruloplasmin [22], with both latter proteins also included in the list of predictive proteins. The complexes of lipoprotein (A-I) with these ions appear to contain transferrin and ceruloplasmin and to inhibit oxidation of LDL in vitro. Since it is believed that LDL oxidation is a key element in the atherosclerotic process, high excretion of APOA1 and transferrin in urine of the steroid sensitive patients may reflect higher content of these proteins in serum of these patients, which suggests that steroid sensitive group might have milder sclerosis potential. Comparison of sclerosis status in Table 1 supports this hypothesis (with 100% sensitivity and 75% specificity), the only exception being patient 9 whose biopsy might have been taken from a non-sclerotic part.

MXRA8 (Matrix-remodeling protein 8) was the most significantly underrepresented protein (fold change 1.5) in steroid sensitive patients. Matrix-remodeling proteins are believed to associate with the fibrosis process in heart disease [23]. Significant underrepresentation of MXRA8 may reflect the higher prevalence of interstitial fibrosis (IF) in non-responders (Table 1). Indeed, three out of four non-responders have IF >10%, while four out of six responders have IF <10%.

Among the proteins in the predictive list (Additional file 3) are CUBN, IGHG1, CERU, TFRE and A2GL, which are urinary glycoproteins associated with chronic kidney disease [24]. CUBN (Cubilin) is highly expressed in the renal proximal tubule and the visceral yolk sac [25] and other tissues. Cubilin was shown to be an endocytic receptor for apolipoprotein A-I (apoA-I)/high density lipoprotein (HDL), mediating uptake of HDL in the kidney and visceral endothelium [26,27]. Other cubilin ligands include albumin, transferrin, immunoglobulin light chains, vitamin D-binding protein, myoglobin, galectin-3 and Clara cell secretory protein [28]. Underrepresentation of CUBN in our data set which correlates with overrepresentation of APOA1 and TRFE (as ligands for CUBN) leads us to the hypothesis that the capacity of kidney and other CUBN-expressing tissues for APOA-1 uptake and binding to TRFE is decreased in the steroid sensitive compared to steroid resistant group. Elevated excretion level of IGHG1 (a ligand for CUBN) also may be linked to underrepresentation of CUBN.

Four of the predictive proteins (CLUS, A1AG2, AACT and TRFE) were enriched in the GO process “acute inflammatory response” as the most significant biological process according to DAVID. Implication of inflammation in pathogenesis of FSGS has been found in some studies [29,30]. Scandrett et al. in 1995 reported the dependence of glomerulosclerosis following antiglomerular basement membrane nephritis to the degree of acute inflammatory injury [31]. Tsai et al. have recently investigated the anti-inflammatory effect of a traditional drug in FSGS treatment [29].

CLUS (Clusterin) is a ubiquitous protein and is a component of the high-density lipoprotein (HDL) complex with putative functions in the transport of lipids, apoptosis, protection of cells from stress and, most importantly, in the regulation of complement activity [32]. Previous studies indicate that clusterin also may have some pathogenic role in FSGS [32]. In fact, clusterin has been recognized as one of the most active physiological inhibitors [33] of the hitherto uncharacterized circulating plasma factor that is the putative cause of the disease [34,35]. Thus, its deficiency should negatively affect proteinuria in this condition. Therefore, underrepresentation of urinary clusterin in steroid sensitive patients may explain the higher level of proteinuria in these patients (mean = 4038 mg/day) compared to the steroid resistant group (mean = 3259 mg/day).

A1AG2 (Alpha-1-acid glycoprotein 2) is an acute-phase protein; its blood concentration is therefore significantly increased in inflammatory states [36]. This protein has been reported as a urinary biomarker of glomerular disease [37]. In cancer studies, increased α_1_-acid glycoprotein levels have been interpreted as the presence of inflammation that complicated the treatment regime [38,39]. Slight overrepresentation of A1AG2 in the urine of steroid resistant patients may reflect its higher concentration in blood, which in turn may be due to a more severe inflammatory status of these patients. The latter suggestion is supported by the enrichment of the “acute inflammatory response” process in that patient group compared to steroid sensitive patients.

AACT (alpha-1-antichymotrypsin) is a glycoprotein in plasma which is primarily synthesized in hepatocytes and acts as a protease inhibitor [40]. The expression of α1-antichymotrypsin in hepatic cells is known to be enhanced by glucocorticoids (steroids) [40]. Overrepresentation of this protein in the urine of steroid sensitive patients is thus an expected result, reflecting correlation between this protein and steroids.

Urine excreted AACT (α1-antichymotrypsin) has been found before in kidney disease and suggested as a potential new urinary biomarker for diagnosing allograft rejection after renal transplantation [41].

PGBM, also known as perlecan or endorepellin, is one of the components of basement membrane of various tissues, especially glomeruli in kidney [42,43]. This is a key molecule in the charge-selective moiety of glomerular filtration [44,45]. Rienstra et al. reported correlation of perlecan expression with severity of interstitial fibrosis and also sclerotic lesions of FGS [46]. Increased excretion of this protein in responders may reflect different status of glomerular filtration in this group in comparison with non-responders.

ACTG (gamma-actin) is the monomeric component of the two-stranded helical structural filament, filamentous actin (F-actin) found in cytoplasm. Gamma-actin binds with numerous other costameric proteins, including talin and vinculin [47]. Foot process effacement is related to derangements in podocyte actin cytoskeleton and glucocorticoids (steroids) ameliorate proteinuria by stabilization of actin filaments [48]. Therefore, overrepresentation of ACTG in steroid sensitive patients may explain effectiveness in these patients of steroid drugs that target ACTG.

A1BG (Alpha-1B-glycoprotein) is a plasma glycoprotein with homology to the immunoglobulin supergene family [49]. Implication of this protein in responsiveness to steroid drugs reported by Pyaphanee et al. [50] and Huang et al. [51]. Underrepresentation of this protein in steroid sensitive patients in our data set is thus consistent with previous findings.

## Conclusion

In conclusion, we discovered a panel of noninvasive urine biomarkers using proteomics tools that are potentially predictive of an efficient therapeutic strategy. Altogether, 21 protein candidates were identified with the most drastic fold change exhibited by APOA-1 and MXRA8. GO-term enrichment analysis confirmed that acute inflammatory response is the main biological process responsible for such differentiation. Further experiments are needed to validate these findings and create a reliable predictive model for steroid therapy responsiveness.

## Abbreviations

FSGS: Focal segmental glomerulosclerosis; eGFR: Estimated glomerular filtration rate; CKD-EPI: Chronic kidney disease epidemiology collaboration; BCA: Bicinchoninic acid; DTT: Dithiothreitol; IAA: Iodoacetamide; FDR: False discovery rate; PCA: Principal component analysis; PC: Principal component; OPLS-DA: Orthogonal projection to latent structures discriminant analysis; ROC: Receiver operating characteristic; GO: Gene ontology; AUC: Area under curve.

## Competing interests

The authors declare that they have no competing interests.

## Authors’ contributions

SK: carried out the experiment, analyzed the data and drafted the manuscript. MN: Participated in the design of the study, interpreting the data and sample collection and performing biopsy. DR: participated in performing the experiment. SS: participated in sample collection, performing biopsy and interpreting the data. MT: participated in sample collection and primary preparation. HY: participated in analyzing the data and assisted in statistical part and preparing the graphs in the manuscript. RZ: Participated in the design of the study and its coordination, data analysis and helped to draft the manuscript. All the authors read and approved the final manuscript.

## Pre-publication history

The pre-publication history for this paper can be accessed here:

http://www.biomedcentral.com/1471-2369/15/141/prepub

## Supplementary Material

Additional file 1 All quantified proteins using label-free quantification method in the urine of steroid sensitive and steroid resistant patients. Click here for file

Additional file 2 List of quantified proteins after preprocessing procedure. Click here for file

Additional file 3 Predictive biomarker candidates for responsiveness to steroid therapy in FSGS patients. Click here for file

Additional file 4 Gene ontology enrichment analysis of predictive OPLS-DA model using DAVID software. Click here for file
