# Supplementary material for: Predictive urinary biomarkers for steroid-resistant and steroid-sensitive focal segmental glomerulosclerosis using high resolution mass spectrometry and multivariate statistical analysis
Source: BMC Nephrol. 2014 Sep 2;15:141. doi: 10.1186/1471-2369-15-141 (PMC4236676; doi:10.1186/1471-2369-15-141)
Supplement: Additional file 3 — Predictive biomarker candidates for responsiveness to steroid therapy in FSGS patients. [file 1471-2369-15-141-S3.docx]

**Additional file 3:** Predictive biomarker candidates for responsiveness to steroid therapy in FSGS patients.

| **Protein ID** | **Protein Name** | **Biological Process** | **Cellular Component** | **Molecular Function** | **Fold Change (SS/SR)** | **Up/Down regulation** |
| --- | --- | --- | --- | --- | --- | --- |
| APOA1 | Apolipoprotein A-I | negative regulation of cytokine secretion involved in immune response/negative regulation of inflammatory response | spherical high-density lipoprotein particle/secretory granule | high-density lipoprotein particle binding | 3.15 | ↑ |
| PGRP2 | N-acetylmuramoyl-L-alanine amidase | negative regulation of natural killer cell differentiation involved in immune response | extracellular region | N-acetylmuramoyl-L-alanine amidase activity/peptidoglycan receptor activity/zinc ion binding | 2.29 | ↑ |
| ACTG | Gamma-actin | response to calcium ion/ adherens junction organization | cytosol | ATP binding/structural constituent of cytoskeleton | 1.74 | ↑ |
| FBLN3 | EGF-containing fibulin-like extracellular matrix protein 1 | peptidyl-tyrosine phosphorylation/visual perception | proteinaceous extracellular matrix | calcium ion binding/epidermal growth factor-activated receptor activity | 1.62 | ↑ |
| PGBM | Basement membrane-specific heparan sulfate proteoglycan core protein | [Angiogenesis](http://www.uniprot.org/keywords/KW-0037)/[lipoprotein metabolic process](http://www.ebi.ac.uk/QuickGO/GTerm?id=GO:0042157) | [Basement membrane](http://www.uniprot.org/keywords/KW-0084)/Extracellular matrix/Secreted | Metal ion binding | 1.55 | ↑ |
| AACT | Alpha-1-antichymotrypsin | [inflammatory response](http://www.ebi.ac.uk/QuickGO/GTerm?id=GO:0006954)/ [regulation of lipid metabolic process](http://www.ebi.ac.uk/QuickGO/GTerm?id=GO:0019216) | extracellular region | [serine-type endopeptidase inhibitor activity](http://www.ebi.ac.uk/QuickGO/GTerm?id=GO:0004867) | 1.49 | ↑ |
| IGHG1 | Ig gamma-1 chain C region | [complement activation, classical pathway](http://www.ebi.ac.uk/QuickGO/GTerm?id=GO:0006958) | [extracellular region](http://www.ebi.ac.uk/QuickGO/GTerm?id=GO:0005576)/membrane | [antigen binding](http://www.ebi.ac.uk/QuickGO/GTerm?id=GO:0003823) | 1.25 | ↑ |
| TRFE | Transferrin | [cellular iron ion homeostasis](http://www.ebi.ac.uk/QuickGO/GTerm?id=GO:0006879)/ Ion transport | extracellular region/Apical and basal plasma membrane | [ferric iron binding](http://www.ebi.ac.uk/QuickGO/GTerm?id=GO:0008199) | 1.14 | ↑ |
| YIPF3 | Killer lineage protein 1 | [cell differentiation](http://www.ebi.ac.uk/QuickGO/GTerm?id=GO:0030154) | memberane | May play a role in hematopoiesis | 1.12 | ↓ |
| A1AG2 | Alpha-1-acid glycoprotein 2 | [acute-phase response](http://www.ebi.ac.uk/QuickGO/GTerm?id=GO:0006953)/[regulation of immune system process](http://www.ebi.ac.uk/QuickGO/GTerm?id=GO:0002682) | [extracellular space](http://www.ebi.ac.uk/QuickGO/GTerm?id=GO:0005615) | Appears to function in modulating the activity of the immune system during the acute-phase reaction/ transport protein in the blood stream | 1.15 | ↓ |
| THBG | Thyroxine-binding globulin | [response to corticosterone stimulus](http://www.ebi.ac.uk/QuickGO/GTerm?id=GO:0051412)/[regulation of proteolysis](http://www.ebi.ac.uk/QuickGO/GTerm?id=GO:0030162) | [extracellular space](http://www.ebi.ac.uk/QuickGO/GTerm?id=GO:0005615) | [serine-type endopeptidase inhibitor activity](http://www.ebi.ac.uk/QuickGO/GTerm?id=GO:0004867) | 1.17 | ↓ |
| A1BG | Alpha-1B-glycoprotein | Immune system | [Secreted](http://www.uniprot.org/keywords/KW-0964) | Act like immunoglobulin | 1.22 | ↓ |
| A2GL | Leucine-rich alpha-2-glycoprotein | [brown fat cell differentiation](http://www.ebi.ac.uk/QuickGO/GTerm?id=GO:0050873) | [extracellular space](http://www.ebi.ac.uk/QuickGO/GTerm?id=GO:0005615) | involved in protein-protein interaction, signal transduction, and cell adhesion | 1.23 | ↓ |
| ANAG | Alpha-N-acetylglucosaminidase | [glycosaminoglycan catabolic process](http://www.ebi.ac.uk/QuickGO/GTerm?id=GO:0006027) | [lysosomal lumen](http://www.ebi.ac.uk/QuickGO/GTerm?id=GO:0043202) | [alpha-N-acetylglucosaminidase activity](http://www.ebi.ac.uk/QuickGO/GTerm?id=GO:0004561) | 1.31 | ↓ |
| S10A9 | Calgranulin-B | [chronic inflammatory response](http://www.ebi.ac.uk/QuickGO/GTerm?id=GO:0002544) | extracellular region/[cytosol](http://www.ebi.ac.uk/QuickGO/GTerm?id=GO:0005829)/[plasma membrane](http://www.ebi.ac.uk/QuickGO/GTerm?id=GO:0005886) | [Toll-like receptor 4 binding](http://www.ebi.ac.uk/QuickGO/GTerm?id=GO:0035662)/zinc & calcium ion binding | 1.35 | ↓ |
| CUBN | Cubilin | [lipoprotein metabolic process](http://www.ebi.ac.uk/QuickGO/GTerm?id=GO:0042157)/[receptor-mediated endocytosis](http://www.ebi.ac.uk/QuickGO/GTerm?id=GO:0006898) | [brush border membrane](http://www.ebi.ac.uk/QuickGO/GTerm?id=GO:0031526)/[Golgi apparatus](http://www.ebi.ac.uk/QuickGO/GTerm?id=GO:0005794)/Lysosom | [cobalamin & calcium binding](http://www.ebi.ac.uk/QuickGO/GTerm?id=GO:0031419)/[receptor activity](http://www.ebi.ac.uk/QuickGO/GTerm?id=GO:0004872) | 1.38 | ↓ |
| TITIN | Connectin | [regulation of protein kinase activity](http://www.ebi.ac.uk/QuickGO/GTerm?id=GO:0045859)/[response to calcium ion](http://www.ebi.ac.uk/QuickGO/GTerm?id=GO:0051592) | extracellular region/[Golgi apparatus](http://www.ebi.ac.uk/QuickGO/GTerm?id=GO:0005794)/cytosol | [protein serine & threonine & tyrosin kinase activity](http://www.ebi.ac.uk/QuickGO/GTerm?id=GO:0004674)/calcium ion binding | 1.44 | ↓ |
| AMPN | Aminopeptidase N | [cell differentiation](http://www.ebi.ac.uk/QuickGO/GTerm?id=GO:0030154)/[proteolysis](http://www.ebi.ac.uk/QuickGO/GTerm?id=GO:0006508) | Cytosis/plasma membrane | [aminopeptidase activity](http://www.ebi.ac.uk/QuickGO/GTerm?id=GO:0004177)/receptor activity/zinc ion binding | 1.46 | ↓ |
| CLUS | Clusterin | [complement activation, classical pathway](http://www.ebi.ac.uk/QuickGO/GTerm?id=GO:0006958)/[lipid metabolic process](http://www.ebi.ac.uk/QuickGO/GTerm?id=GO:0006629) | [cytosol](http://www.ebi.ac.uk/QuickGO/GTerm?id=GO:0005829)/[endoplasmic reticulum](http://www.ebi.ac.uk/QuickGO/GTerm?id=GO:0005783)/[mitochondrion](http://www.ebi.ac.uk/QuickGO/GTerm?id=GO:0005739)/[nucleus](http://www.ebi.ac.uk/QuickGO/GTerm?id=GO:0005634) | [ubiquitin protein ligase binding](http://www.ebi.ac.uk/QuickGO/GTerm?id=GO:0031625) | 1.46 | ↓ |
| IPSP | Plasma serine protease inhibitor | [negative regulation of proteolysis](http://www.ebi.ac.uk/QuickGO/GTerm?id=GO:0045861)/lipid transport | [extracellular space](http://www.ebi.ac.uk/QuickGO/GTerm?id=GO:0005615) | [serine-type endopeptidase inhibitor activity](http://www.ebi.ac.uk/QuickGO/GTerm?id=GO:0004867)/[phosphatidylcholine binding](http://www.ebi.ac.uk/QuickGO/GTerm?id=GO:0031210) | 1.47 | ↓ |
| MXRA8 | Matrix-remodeling-associated protein 8 | Fibrosis process [23] | [Membrane](http://www.uniprot.org/keywords/KW-0472) | May play a role in the maturation and maintenance of blood-brain barrier | 1.49 | ↓ |

(SS: Steroid sensitive, SR: Steroid resistant)
